# Self‐Healing Ionogel‐Enabled Self‐Healing and Wide‐Temperature Flexible Zinc‐Air Batteries with Ultra‐Long Cycling Lives

**DOI:** 10.1002/advs.202402193

**Published:** 2024-04-03

**Authors:** Hongli Li, Fuchang Xu, Yang Li, Junqi Sun

**Affiliations:** ^1^ State Key Laboratory of Supramolecular Structure and Materials College of Chemistry Jilin University Changchun 130012 P. R. China

**Keywords:** environmental and electrochemical stability, flexible zinc‐air batteries, self‐healing ionogels, self‐healing materials, wide operating temperature

## Abstract

Hydrogel‐based zinc‐air batteries (ZABs) are promising flexible rechargeable batteries. However, the practical application of hydrogel‐based ZABs is limited by their short service life, narrow operating temperature range, and repair difficulty. Herein, a self‐healing ionogel is synthesized by the photopolymerization of acrylamide and poly(ethylene glycol) monomethyl ether acrylate in 1‐ethyl‐3‐methylimidazolium dicyanamide with zinc acetate dihydrate and first used as an electrolyte to fabricate self‐healing ZABs. The obtained self‐healing ionogel has a wide operating temperature range, good environmental and electrochemical stability, high ionic conductivity, satisfactory mechanical strength, repeatable and efficient self‐healing properties enabled by the reversibility of hydrogen bonding, and the ability to inhibit the production of dendrites and by‐products. Notably, the self‐healing ionogel has the highest ionic conductivity and toughness compared to other reported self‐healing ionogels. The prepared self‐healing ionogel is used to assemble self‐healing flexible ZABs with a wide operating temperature range. These ZABs have ultra‐long cycling lives and excellent stability under harsh conditions. After being damaged, the ZABs can repeatedly self‐heal to recover their battery performance, providing a long‐lasting and reliable power supply for wearable devices. This work opens new opportunities for the development of electrolytes for ZABs.

## Graphical abstract

Self‐healing ionogels with high ionic conductivity and good mechanical properties are synthesized and used to fabricate self‐healing and wide‐temperature flexible ZABs with ultra‐long cycling lives for the first time. These ZABs have excellent stability under harsh conditions. After being damaged, the ZABs can repeatedly self‐heal to recover their battery performance, providing a long‐lasting and reliable power supply for wearable devices.

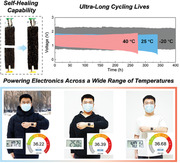

## 1 Introduction

Flexible rechargeable batteries with high energy density and long service life have attracted widespread attention in recent years due to the rapid popularization of wearable flexible devices.^[^ ^1^ ^]^ Hydrogel‐based zinc‐air batteries (ZABs) are one of the most promising candidates for next‐generation flexible rechargeable batteries due to their high theoretical energy density, good safety, environmental friendliness, low cost, and leak‐proof electrolytes.^[^ ^2^, ^3^ ^]^ However, although ZABs prepared with hydrogels containing different polymers and additives have been demonstrated, the practical implementation of hydrogel‐based ZABs is still limited by their inherent disadvantages.^[^ ^4^, ^5^ ^]^ 1) Hydrogel‐based ZABs suffer from short cycling lives.^[^ ^6^ ^]^ The semi‐open structure of these ZABs allows the water in the hydrogels to continuously dissipate into the air, resulting in a drastic decrease in the ionic conductivity of the hydrogels over time. Consequently, most hydrogel‐based ZABs have cycling lives of a few tens of hours at room temperature. 2) Hydrogel‐based ZABs operate within a narrow temperature range.^[^ ^7^ ^]^ Hydrogel electrolytes rapidly dehydrate at elevated temperatures and freeze at sub‐zero temperatures, ultimately resulting in battery failure.^[^ ^8^ ^]^ 3) The poor electrochemical stability of some hydrogel electrolytes can negatively impact the stability of the zinc anode.^[^ ^9^ ^]^ Therefore, these hydrogel‐based ZABs have shorter cycling lives. 4) The repeated deformation of hydrogel‐based ZABs can rupture the hydrogel electrolytes, leading to a decline in battery performance.^[^ ^10^ ^]^ Therefore, to prepare flexible ZABs capable of stable and long‐term operation across a wide range of temperatures, it is essential to explore novel electrolytes that are non‐volatile, have good environmental and electrochemical stability, have good ionic conductivity, and exhibit self‐healing properties.

Ionic liquids (ILs) are a class of room‐temperature molten salts composed of organic cations and inorganic anions.^[^ ^11^ ^]^ As next‐generation novel green solvents with promising application prospects in materials science, catalysis, purification, separation, and electrochemistry, ILs are praised for their high ionic conductivity, good chemical and thermal stability, low freezing temperature, wide electrochemical window, and non‐volatility.^[^ ^12^, ^13^, ^14^ ^]^ Recently, self‐healing ionogels, which are IL‐based novel materials, have been fabricated by immobilizing ILs in polymer networks cross‐linked by dynamic covalent bonds (disulfide bonds, boronic esters, and imine bonds) or noncovalent bonds (hydrogen bonds, coordinate bonds, electrostatic interaction, host‐guest recognition, and *π*–*π* interaction).^[^ ^15^, ^16^, ^17^, ^18^, ^19^, ^20^, ^21^ ^]^ In addition to inheriting the advantages of ILs, self‐healing ionogels possess the capability to heal damage spontaneously or in response to external stimuli such as light,^[^ ^22^ ^]^ solvent,^[^ ^23^ ^]^ and heat.^[^ ^24^ ^]^ Once damaged, self‐healing ionogels can reconstruct their polymer network at the injured site, to reestablish their original structural and functional integrity. This reconstruction is enabled by their reversible covalent and noncovalent bonds.^[^ ^25^, ^26^ ^]^ Over the past decade, various self‐healing ionogels with tunable ionic conductivity, sensitivity, thermal response, and gas adsorption properties have been created by adjusting the composition of ILs and polymers, and these ionogels have shown promise for use in sensors,^[^ ^27^, ^28^ ^]^ smart windows,^[^ ^29^ ^]^ and gas separation.^[^ ^30^ ^]^ In particular, self‐healing ionogels have shown great promise as a substitute for hydrogels in the field of electrolyte materials due to their non‐volatility, environmental and electrochemical stability, and self‐healing ability.^[^ ^31^, ^32^, ^33^, ^34^ ^]^ For instance, Yan and coworkers reported the fabrication of a self‐healing ionogel with electrical sensing capabilities through the free radical polymerization of N, N‐dimethylacrylamide and poly(1‐acrylamido‐2‐methylpropane sulfonic acid) microspheres in 1‐ethyl‐3‐methylimidazolium bis(trifluoromethanesulfonyl)imide.^[^ ^17^ ^]^ The resulting ionogel possessed self‐healing properties due to its reversible hydrogen bonds and ionic bonds. In addition, compared to hydrogels, which exhibit reduced ionic conductivity over time because of water evaporation, the resulting ionogel exhibited stable ionic conductivity and electrical sensing performance.

The use of self‐healing ionogels as electrolytes in the preparation of flexible ZABs shows excellent potential for solving the aforementioned issues of hydrogel‐based ZABs, including their short cycling life, narrow operating temperature range, and repair difficulty. However, this strategy has not been successfully employed yet because existing self‐healing ionogels have some shortcomings that make them unsuitable for use in ZABs. Specifically, most reported self‐healing ionogels have low ionic conductivities.^[^ ^35^ ^]^ This is mainly because the ILs contained in self‐healing ionogels have low ionic conductivity or because the poor compatibility between the ILs and polymer networks in self‐healing ionogels leads to low IL content. Moreover, there exists a trade‐off between the ionic conductivity and mechanical strength of self‐healing ionogels.^[^ ^36^, ^37^ ^]^ Enhancing the ionic conductivity of ionogels by increasing their IL content will reduce their mechanical strength, making them more susceptible to damage. Therefore, preparing self‐healing ionogels that can overcome this trade‐off to simultaneously achieve high ionic conductivity and good mechanical strength would be of significant research and application value.

Herein, a self‐healing ionogel (named PAM‐PEGMA‐IL) with high ionic conductivity and good mechanical properties was synthesized and used to fabricate self‐healing and wide‐temperature flexible ZABs (named SWF‐ZABs) with long cycling lives for the first time. The PAM‐PEGMA‐IL ionogel was synthesized by the in‐situ polymerization of acrylamide (AM) and poly(ethylene glycol) monomethyl ether acrylate (PEGMA) in 1‐ethyl‐3‐methylimidazolium dicyanamide ([Emim][DCA]) with zinc acetate dihydrate. The abundant reversible hydrogen bonds formed between the amide groups in the PAM‐PEGMA‐IL ionogel strengthened its mechanical properties and provided efficient and repeatable self‐healing properties, even at sub‐zero temperatures. Due to the good compatibility between the PEG segments and [Emim][DCA], the PAM‐PEGMA‐IL ionogel contained up to 80 wt.% [Emim][DCA]. The rational composition design of the PAM‐PEGMA‐IL ionogel provided it with high ionic conductivity, good mechanical properties, a wide operating temperature range, excellent self‐healing efficiency, a high decomposition voltage, excellent stability in harsh environments, and the ability to inhibit by‐products and dendrite growth. Consequently, the obtained SWF‐ZABs had ultra‐long cycling lives of 400, 340, and 275 h at −20, 25, and 40 °C, which are higher than the cycling lives of other reported self‐healing hydrogel‐based ZABs. Moreover, the SWF‐ZABs successfully maintained their battery performance after storage at −20 or 40 °C in air and 25 °C in vacuum for 10 days, which is not possible with hydrogel‐based ZABs. After being damaged, the SWF‐ZABs were able to repeatedly and fully self‐heal, recovering their battery performance. This significantly increased their service life and reliability. The SWF‐ZABs were also integrated into various wearable electronic devices (e.g., digital watch and patch thermometer), acting as a reliable power source under a wide temperature range.

## 2 Results and Discussion

### 2.1 Synthesis and Characterization of PAM‐PEGMA‐IL Ionogels

**Figure 1:**
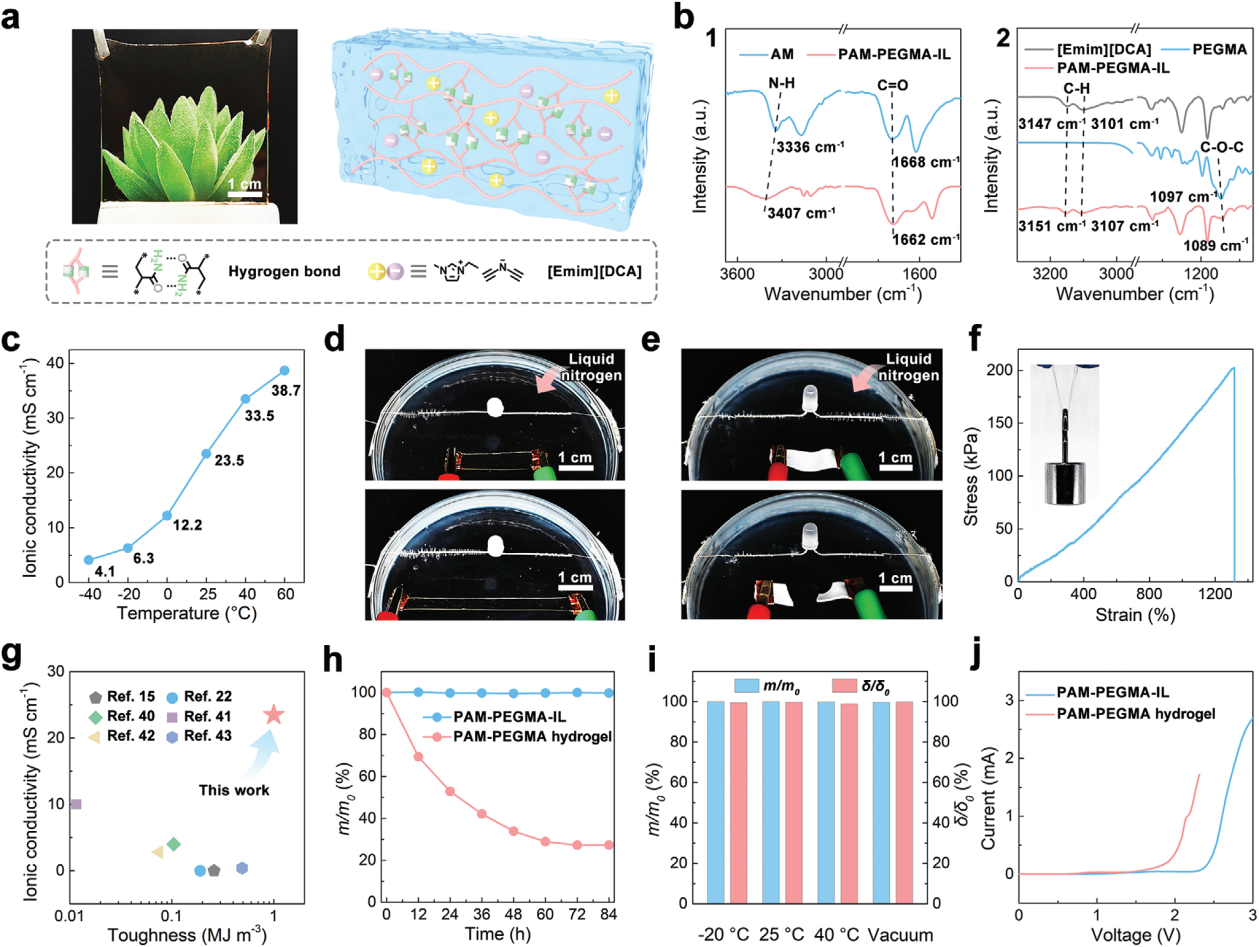
a) Photograph of the PAM‐PEGMA‐IL ionogel and schematic illustration of its internal structure. b) FTIR spectra of (1) AM and the PAM‐PEGMA‐IL ionogel and (2) [Emim][DCA], PEGMA, and the PAM‐PEGMA‐IL ionogel. c) Temperature‐dependent ionic conductivity of the PAM‐PEGMA‐IL ionogel. d) Sequential photographs showing the PAM‐PEGMA‐IL ionogel being stretched to 200% strain at −60 °C while maintaining conductive properties. e) Sequential photographs showing the PAM‐PEGMA hydrogel freezing and breaking under slight deformation at −60 °C. f) Stress–strain curves of the PAM‐PEGMA‐IL ionogel. Inset: Photograph of the PAM‐PEGMA‐IL ionogel lifting a 100 g weight. g) Ashby plot of the ionic conductivity and toughness of the PAM‐PEGMA‐IL ionogel and other reported room temperature self‐healing ionogels containing 80 wt.% IL. h) Weight changes (*m*/*m*_0_) of the PAM‐PEGMA‐IL ionogel and the PAM‐PEGMA hydrogel at 25 °C. i) Weight changes (*m*/*m*_0_) and ionic conductivity changes (*δ*/*δ*_0_) of the PAM‐PEGMA‐IL ionogel after storage −20, 25, and 40 °C in air and at 25 °C in vacuum. j) Decomposition voltages of the PAM‐PEGMA‐IL ionogel and the PAM‐PEGMA hydrogel.

A promising self‐healing ionogel used as an electrolyte in a flexible ZAB should meet the following criteria: 1) The polymer network of the ionogel should have good compatibility with the IL to maximize IL loading. 2) The IL contained within the ionogel should have a low freezing point, high decomposition temperature, good electrothermal stability, good environmental stability, and high ionic conductivity. 3) The noncovalent bonds within the ionogel should have good electrochemical stability and reversibility, ensuring good electrochemical stability and self‐healing properties. After a literature survey and series of pre‐experiments, in this study, hydrogen bonding cross‐linked self‐healing ionogels that meet these abovementioned criteria were synthesized via the 1‐hydroxycyclohexyl phenyl ketone‐initiated photopolymerization of AM and PEGMA in [Emim][DCA] with zinc acetate dihydrate under UV irradiation. AM and PEGMA were chosen due to the formation of hydrogen bonds between amide groups and the high compatibility of PEG segments with most ILs. **Figures** **1**a and S1 (Supporting Information) indicate the obtained PAM‐PEGMA‐IL ionogel was transparent, showing the uniform dispersion of [Emim][DCA] and zinc acetate dihydrate without any leakage or precipitation. Fourier transform infrared (FTIR) spectroscopy was used to investigate the interactions within the PAM‐PEGMA‐IL ionogel. Compared to the AM spectrum, the N─H stretching vibration peak in the PAM‐PEGMA‐IL ionogel spectrum (3407 cm^−1^) was observed to shift to a higher wavenumber (3336 cm^−1^), as shown in Figure 1b1 and Figure S2 (Supporting Information). Furthermore, the C═O stretching vibration peak in the PAM‐PEGMA‐IL ionogel spectrum (1662 cm^−1^) was shifted to a lower wavenumber (1668 cm^−1^) compared to AM. This confirmed that the PAM‐PEGMA‐IL ionogel was cross‐linked by abundant reversible hydrogen bonds (Figure 1a). The presence of these hydrogen bonds ensured that the ionogel exhibited good mechanical strength and electrochemical stability as well as self‐healing properties. As shown in Figure 1b2 and Figure S2 (Supporting Information), the C─H stretching vibration peak of the imidazolium rings in the PAM‐PEGMA‐IL ionogel spectrum (3107 and 3151 cm^−1^) was shifted to a higher wavenumber compared to the [Emim][DCA] spectrum (3101 and 3147 cm^−1^). Meanwhile, the C─O─C stretching vibration peak in the PAM‐PEGMA‐IL ionogel spectrum (1089 cm^−1^) was shifted to a lower wavenumber compared to the PEGMA spectrum (1097 cm^−1^), suggesting the presence of hydrogen bonds between the ether groups of PEG segments and the imidazolium rings of [Emim][DCA]. This improved the compatibility between [Emim][DCA] and polymer networks, enabling the PAM‐PEGMA‐IL ionogel to contain up to 80 wt.% [Emim][DCA] without leakage.

[Emim][DCA] has a room‐temperature ionic conductivity of 28 mS cm^−1^, freezing temperature of −24 °C, and decomposition temperature of 284 °C (Figure S3, Supporting Information).^[^ ^38^ ^]^ Therefore, the high loading of [Emim][DCA] provided the PAM‐PEGMA‐IL ionogel with high ionic conductivity and a wide operating temperature range. Figure 1c shows that the ionic conductivities of the PAM‐PEGMA‐IL ionogel at −40, 25, and 60 °C were 4.1, 23.5, and 38.7 mS cm^−1^, respectively. The differential scanning calorimetry (DSC) curve in Figure S4 (Supporting Information) shows that the glass transition temperature and freezing temperature of the PAM‐PEGMA‐IL ionogel were lower than −75 °C, which was the lowest temperature measurable by our instrument. The freezing temperature of the PAM‐PEGMA‐IL ionogel was significantly lower than that of [Emim][DCA]. This was due to the hydrogen bonding between the polymer network and the [Emim][DCA], which weakened the ion–ion interactions between [Emim] and [DCA] and hindered the crystallization of [Emim][DCA].^[^ ^38^, ^39^ ^]^ Therefore, the PAM‐PEGMA‐IL ionogel was able to maintain its conductivity and elasticity at a height of 1 cm above liquid nitrogen (Figure 1d; Movie S1, Supporting Information), where the temperature was ≈−60 °C. In contrast, a PAM‐PEGMA hydrogel, which was fabricated by replacing [Emim][DCA] in the feedstock with water, froze and broke under slight deformation when placed at the same height above liquid nitrogen (Figure 1e; Movie S2, Supporting Information). Thermogravimetric analysis (TGA) and rheological measurements revealed that the PAM‐PEGMA‐IL ionogel had a decomposition temperature and solid‐to‐liquid transition temperature of 223 and 65 °C, respectively (Figures S3 and S5, Supporting Information). These results indicate that the PAM‐PEGMA‐IL ionogel had an operating temperature range of −75–65 °C, showing promise for use in ZABs.

A thin strip of the PAM‐PEGMA‐IL ionogel with dimensions of 30 mm × 10 mm × 0.5 mm was capable of lifting a 100 g weight without breaking (Figure 1f inset). This was 500 times the weight of the ionogel sample, indicating its good mechanical strength. Tensile tests were conducted at a stretching speed of 50 mm min^−1^ to measure the mechanical properties of the PAM‐PEGMA‐IL ionogel. The stress‐strain curve in Figure 1f shows that the PAM‐PEGMA‐IL ionogel exhibited typical elastomeric characteristics. The tensile strength, strain at break, and toughness of the PAM‐PEGMA‐IL ionogel were 202 kPa, 1318%, and 1.19 MJ m^−3^, respectively. Notably, the mass ratio of AM to PEGMA in the feedstock was consistently maintained at 7:3 throughout this study. Pre‐experiments indicated that reducing the AM content hindered the formation of the ionogel due to insufficient hydrogen bond cross‐linking (Figure S6a, Supporting Information). Conversely, an excessively high AM content led to IL leakage due to the reduced compatibility of the polymer network with the IL (Figure S6b, Supporting Information). The rational compositional design of PAM‐PEGMA‐IL provided this ionogel with the highest ionic conductivity and toughness compared to other reported room‐temperature self‐healing ionogels containing 80 wt.% IL (Figure 1g),^[^ ^15^, ^22^, ^40^, ^41^, ^42^, ^43^ ^]^ which was beneficial for their use as electrolytes.

The gradual water loss from the hydrogel electrolytes of hydrogel‐based ZABs causes a significant decrease in the hydrogel ionic conductivity. Therefore, hydrogel‐based ZABs usually suffer from low efficiencies and short cycling lives. For instance, the weight of the PAM‐PEGMA hydrogel decreased by 73% and its ionic conductivity dropped from 29.3 to 0.005 mS cm^−1^ after storage at 25 °C for 84 h (Figure 1h). In contrast, the PAM‐PEGMA‐IL ionogel did not experience any changes in weight or ionic conductivity after being stored at 25 °C for 84 h. Furthermore, the PAM‐PEGMA‐IL ionogel was able to maintain its weight and ionic conductivity after storage at −20, 25, and 40 °C in air as well as 25 °C in vacuum (relative vacuum pressure = −0.098 MPa) for 10 days (Figure 1i). These results indicate that the PAM‐PEGMA‐IL ionogel had excellent environmental stability, which was attributed to the non‐volatility and thermal stability of [Emim][DCA]. Due to the electrochemical stability of the hydrogen bonds and [Emim][DCA],^[^ ^44^, ^45^ ^]^ the PAM‐PEGMA‐IL ionogel had a decomposition voltage of 2.43 V (Figure 1j). In contrast, the PAM‐PEGMA hydrogel had a decomposition voltage of only 1.61 V, which was due to the 1.23 V potential window of water (Figure 1j).^[^ ^46^ ^]^ The above experiments were conducted under ambient humidity conditions that fluctuated between 30% relative humidity (RH) and 40% RH due to changes in the weather.

### 2.2 Self‐Healing Capability of PAM‐PEGMA‐IL Ionogel

**Figure 2:**
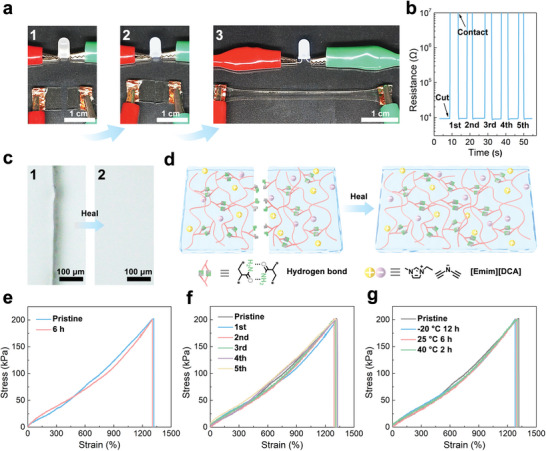
a) Photographs of the cut PAM‐PEGMA‐IL ionogel (1) before and (2) after healing, and (3) the healed PAM‐PEGMA‐IL ionogel being stretched to 400% strain. b) Conductivity recovery of the PAM‐PEGMA‐IL ionogel after different cutting/healing cycles. c) Optical microscopy images of the cut on the PAM‐PEGMA‐IL ionogel before and after healing. d) Schematic of the self‐healing mechanism of the PAM‐PEGMA‐IL ionogel. e) Stress–strain curves of the pristine and healed PAM‐PEGMA‐IL ionogel. f) Stress–strain curves of the PAM‐PEGMA‐IL ionogel after different cutting and healing cycles. g) Stress–strain curves of the cut PAM‐PEGMA‐IL ionogel after healing for different lengths of time at different temperatures.

The self‐healing capability of electrolytes can significantly enhance the stability and lifespan of ZABs. A piece of the PAM‐PEGMA‐IL ionogel with dimensions of 10 mm × 8 mm was cut in half using a scalpel to test its self‐healing capability (**Figure** **2**a1). When the severed ionogel was reconnected, a light‐emitting diode (LED) bulb in the same circuit lit up (Figure 2a2), indicating that the ionogel regained its electrical conductivity. Figure 2b shows the time evolution of the change in resistance that occurred during the cutting and reconnecting processes of the PAM‐PEGMA‐IL ionogel. When the PAM‐PEGMA‐IL ionogel was severed, its resistance increased beyond the upper limit of detection, indicating an open circuit. However, upon reconnecting the separated parts of the PAM‐PEGMA‐IL ionogel, the resistance returned to its initial value within 1 s, further confirming the self‐healing of the ionogel conductivity. Additionally, the wound on the ionogel gradually lightened over time at 25 °C and was invisible to the naked eye after 6 h. Microscopic observation confirmed the complete healing of the wound on the ionogel (Figure 2c). Moreover, the ionogel was able to illuminate an LED bulb while being stretched to 400% of its original length (Figure 2a3). Figure 2d illustrates the self‐healing mechanism of the PAM‐PEGMA‐IL ionogel. The polymer chains diffused and interposed between the wound surfaces of the ionogel, reconstructing the cross‐linked polymer network by reforming the reversible hydrogen bonds.^[^ ^47^ ^]^ Over time, the voids in the wound area were gradually filled by the newly formed polymer networks, ultimately achieving the self‐healing of the ionogel. The self‐healing capability of the PAM‐PEGMA‐IL ionogel was quantitatively assessed through tensile tests. As shown in Figure 2e, the stress‐strain curve of the cut ionogel after 6 h of self‐healing at 25 °C overlapped that of the pristine ionogel, indicating the complete restoration of mechanical properties. Accordingly, the healing efficiency of the PAM‐PEGMA‐IL ionogel, defined as the percentage of the tensile stress of the healed sample to that of a pristine sample, was 99.2%. More importantly, the reversible nature of hydrogen bonds allowed the PAM‐PEGMA‐IL ionogel to fully maintain its mechanical properties after multiple cutting/healing cycles, as shown by the nearly fully superimposable stress‐strain curves of the pristine and repeatedly cut‐healed PAM‐PEGMA‐IL ionogel (Figure 2f). After cutting and healing the same area for five times, the healing efficiency of the PAM‐PEGMA‐IL ionogel was still 99.1% and the conductivity of the ionogel was well maintained (Figure 2b), demonstrating its long‐lasting self‐healing capability. The DSC curve in Figure S4 (Supporting Information) shows that the PAM‐PEGMA‐IL ionogel was viscoelastic above −75 °C, indicating that the ionogel was able to self‐heal even at sub‐zero temperatures. As shown in Figure 2g, the PAM‐PEGMA‐IL ionogel fully regained its mechanical properties after 12 h of healing at −20 °C, with a healing efficiency of 99.3%. Additionally, because the dynamics of hydrogen bonds and the movability of polymer chains are proportional to temperature,^[^ ^48^ ^]^ the PAM‐PEGMA‐IL ionogel fully healed its mechanical properties within 2 h at 40 °C (Figure 2g). These results clearly indicate that the PAM‐PEGMA‐IL ionogel has efficient and long‐lasting self‐healing capabilities over a wide range of temperatures.

### 2.3 Electrochemical Performance of SWF‐ZAB

**Figure 3:**
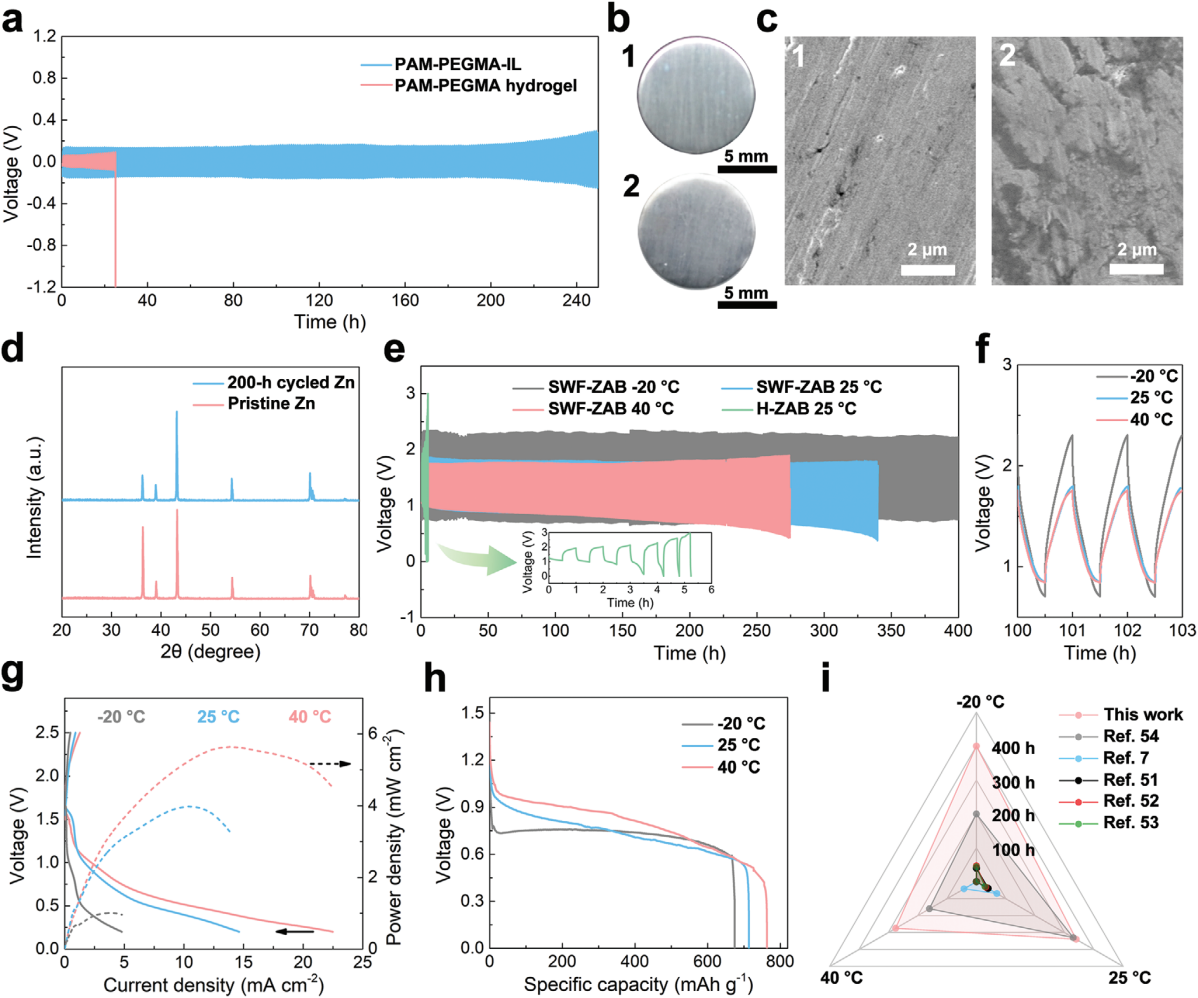
a) Galvanostatic charge/discharge curves of symmetric Zn–Zn cells based on the PAM‐PEGMA‐IL ionogel or PAM‐PEGMA hydrogel at 0.1 mA cm^−2^ with 0.05 mAh cm^−2^. b) Photographs and c) SEM images of (1) pristine Zn foil and (2) Zn foil after cycling for 200 h. d) XRD patterns of the Zn foil before and after cycling for 200 h. e) Galvanostatic charge/discharge curves of SWF‐ZABs at −20, 25, and 40 °C and H‐ZAB at 25 °C with a current density of 0.1 mA cm^−2^. f) Galvanostatic charge/discharge curves of SWF‐ZAB from 100 to 103 h at −20, 25, and 40 °C. g) Polarization curves and corresponding power density curves of SWF‐ZAB at −20, 25, and 40 °C. h) Specific capacities of SWF‐ZAB at −20, 25, and 40 °C. i) Comparison of the cycling lives of SWF‐ZAB at −20, 25, and 40 °C with other reported hydrogel‐based ZABs with wide operating temperatures.

As shown in **Figure** **3**a, Zn–Zn symmetric cells based on the PAM‐PEGMA hydrogel and PAM‐PEGMA‐IL ionogel were tested at 0.1 mA cm^−2^ with 0.05 mAh cm^−2^. The tests were conducted at a temperature of 25 °C, and the humidity of the test environment fluctuated between 30% RH and 40% RH due to changes in the weather. The Zn–Zn symmetric cell based on the PAM‐PEGMA hydrogel exhibited a short cycling life of only 25 h due to the gradual loss of water from the PAM‐PEGMA hydrogel. In contrast, the Zn–Zn symmetric cell based on the PAM‐PEGMA‐IL ionogel exhibited a much longer cycling life of 250 h, benefiting from the non‐volatility of [Emim][DCA]. After 200 h of cycling, the Zn foil removed from the ionogel‐based Zn–Zn symmetric cell still had the same color as pristine Zn foil (Figure 3b). The scanning electron microscopy (SEM) image in Figure 3c1 indicates that the Zn foil was smooth in its pristine state. After 200 h of cycling, the Zn deposition morphology on the Zn foil surface remained uniform, and dendrite growth was not observed (Figure 3c2). Additionally, the X‐ray diffraction (XRD) pattern of the Zn foil cycled for 200 h did not show additional peaks compared to that of the pristine Zn foil, suggesting the absence of any by‐products (Figure 3d). This ability of the PAM‐PEGMA‐IL ionogel to inhibit both dendrite growth and by‐product production was attributed to its high mechanical strength and good electrochemical stability.^[^ ^49^, ^50^ ^]^

Due to its rational material composition design and synthesis, the PAM‐PEGMA‐IL ionogel exhibited a wide operating temperature range, good environmental and electrochemical stability, high ionic conductivity, satisfactory mechanical strength, long‐lasting and efficient self‐healing properties, and the ability to inhibit the production of dendrites and by‐products. These advantages enabled the incorporation of the PAM‐PEGMA‐IL ionogel into SWF‐ZABs capable of operating for extended periods in harsh environments. As a demonstration, SWF‐ZABs were assembled by sandwiching the PAM‐PEGMA‐IL ionogel between a Zn electrode and a nickel foam loaded with commercial Pt/C and RuO_2_ catalysts as an air electrode. Corresponding H‐ZABs were also prepared using the PAM‐PEGMA hydrogel. The cycling performance of both ZABs was tested via galvanostatic charge/discharge measurements at 0.1 mA cm^−2^ with a duration of 1 h per cycle. The test environment had a temperature of 25 °C and a humidity of 30% RH to 40% RH. Figure 3e shows that the SWF‐ZAB had excellent rechargeability, with a cycling life of 340 h and a charge/discharge voltage gap of 0.96 V from the 100th to 103rd h (Figure 3f). In contrast, the H‐ZAB had a short cycling life of only 5 h, and the charge/discharge voltage gap gradually increased to 2.34 V after 4 h, indicating a rapid degradation of battery performance (Figure 3e).^[^ ^3^ ^]^ The cycling life and charge/discharge voltage gap of the SWF‐ZAB were more stable than those of the H‐ZAB due to the good electrochemical and environmental stability of the PAM‐PEGMA‐IL ionogel, which provided a stable reaction environment in the SWF‐ZAB.^[^ ^7^ ^]^ The cycling performance of the SWF‐ZAB was further studied at −20 and 40 °C (Figure 3e,f). The extremely low freezing temperature and good low‐temperature ionic conductivity of the PAM‐PEGMA‐IL ionogel provided the SWF‐ZAB with a cycling life of 400 h and a charge/discharge voltage gap of 1.59 V at −20 °C. In contrast, the PAM‐PEGMA hydrogel in the H‐ZAB froze at −20 °C, resulting in battery failure. Furthermore, at 40 °C, the SWF‐ZAB demonstrated a cycling life of 275 h and a charge/discharge voltage gap of 0.91 V due to the excellent thermal stability of the PAM‐PEGMA‐IL ionogel. In contrast, the H‐ZAB had a cycling life of only 3 h at 40 °C because the PAM‐PEGMA hydrogel rapidly lost water at this high temperature (Figure S7, Supporting Information). As shown in Figure 3g, the SWF‐ZAB exhibited higher discharge polarization voltages, lower charge polarization voltages, and faster charge/discharge kinetics at the same current densities as the temperature increased. Additionally, the maximum power density, specific capacity, and open circuit voltage (OCV) of the SWF‐ZAB also increased with increasing temperature (Figure 3g,h; Figure S8, Supporting Information). This was attributed to the positive correlation between the ionic conductivity of the PAM‐PEGMA‐IL ionogel and temperature.^[^ ^2^ ^]^ The maximum power densities, specific capacities, and OCVs of the SWF‐ZAB at −20, 25, and 40 °C were 1.01 mW cm^−2^, 674 mAh g^−1^ and 1.4675 V, 3.97 mW cm^−2^, 713 mAh g^−1^ and 1.5085 V, and 5.64 mW cm^−2^, 763 mAh g^−1^ and 1.5540 V, respectively. These results indicate that the SWF‐ZAB had a wide operating temperature range, showing good promise for practical applications. Due to the non‐volatility and excellent environmental stability of the PAM‐PEGMA‐IL ionogel, the cycling lives of the SWF‐ZAB at −20, 25, and 40 °C are much higher than those of other reported hydrogel‐based ZABs with wide operating temperatures (Figure 3i).^[^ ^7^, ^51^, ^52^, ^53^, ^54^ ^]^

### 2.4 Application of SWF‐ZAB in Wearable Devices

**Figure 4:**
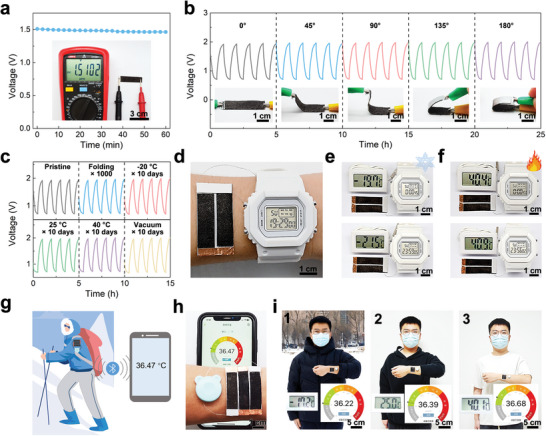
a) OCV of as‐prepared SWF‐ZAB. Inset: photograph showing the OCV of as‐prepared SWF‐ZAB measured using a multimeter. b) Galvanostatic charge/discharge curves of SWF‐ZAB at bending angles of 45°, 90°, 135°, and 180° at a current density of 0.1 mA cm^−1^. c) Galvanostatic charge/discharge curves of SWF‐ZAB in the pristine state, after being folded for 1000 cycles, and after being stored at −20, 25, and 40 °C in air and 25 °C in vacuum for 10 days at a current density of 0.1 mA cm^−1^. d) Photograph showing two SWF‐ZABs connected in series powering a digital watch. e,f) Sequential photographs showing continuous operation of SWF‐ZAB‐powered digital watch for 24 h at (e) −20 °C and (f) 40 °C. g) Schematic diagram showing utilization of SWF‐ZABs to power a patch thermometer to achieve remote temperature monitoring. h) Photograph showing a patch thermometer being powered by three SWF‐ZABs connected in series. This patch thermometer was used to send real‐time body temperature data to a smartphone. For ease of observation, the patch thermometer was attached to the wrist. i) Photographs showing the SWF‐ZAB‐powered patch thermometer sending body temperature data to a smartphone in environments with temperatures of (1) −17.2 °C, (2) 25.0 °C, and (3) 40.1 °C.

To demonstrate the practical application potential of the SWF‐ZAB, several SWF‐ZABs with dimensions of 1 cm × 3 cm were assembled. A single SWF‐ZAB exhibited a stable and high OCV of 1.5102 V under ambient conditions (**Figure** **4**a). In addition, the OCVs of two and three SWF‐ZABs connected in series were twice and three times higher than that of a single SWF‐ZAB, respectively (Figure S9, Supporting Information), indicating the excellent consistency of each SWF‐ZAB. Figure 4b shows the galvanostatic discharge/charge curves of an SWF‐ZAB bent at different angles. Compared to its flat state, the SWF‐ZAB showed similar charging/discharging voltage curves when bent at angles of 45°, 90°, 135°, and 180°, demonstrating its good flexibility. After folding and unfolding 1000 times, the charging/discharging voltage curve of the SWF‐ZAB remained unchanged (Figure 4c), indicating its good deformation stability. Moreover, the charging/discharging voltage curves of the SWF‐ZAB were unchanged after storage at −20, 25, and 40 °C in air as well as 25 °C in vacuum (relative vacuum pressure = −0.098 MPa) for 10 days (Figure 4c). This exceptional environmental stability is uncommon among hydrogel‐based ZABs, providing a reliable guarantee for the long‐term storage and transportation of SWF‐ZABs.

Due to their excellent flexibility, deformation stability, and environmental stability, the SWF‐ZAB showed great potential for use in wearable electronic devices capable of operating in a wide range of temperatures. As shown in Figure 4d, a digital watch was powered by two SWF‐ZABs connected in series. This SWF‐ZAB‐powered digital watch was stably operated for over 24 h at −20 and 40 °C (Figure 4e,f). SWF‐ZABs were also used to power a patch thermometer to achieve the remote real‐time monitoring of human body temperature, useful for avoiding frostbite and heat stroke (Figure 4g). As shown in Figure 4h, three SWF‐ZABs connected in series powered a patch thermometer containing a Bluetooth module that sent real‐time body temperature data to a smartphone. Due to the wide operating temperature range of these SWF‐ZABs, the patch thermometer was able to send the wearer's body temperature to the smartphone at temperatures of −17.2, 25, and 40.1 °C (Figure 4i1–3).

### 2.5 Self‐Healing Capability of SWF‐ZAB

**Figure 5:**
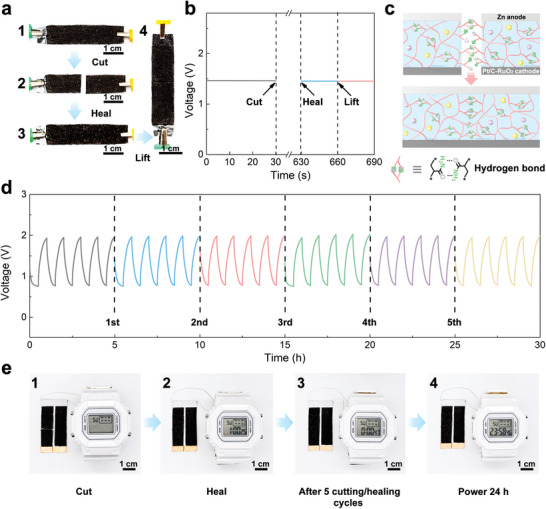
a) Photographs of the (1) pristine, (2) cut, and (3) healed SWF‐ZAB and (4) the healed SWF‐ZAB being lifted from one end without breaking. b) OCV curves of the pristine and healed SWF‐ZAB, and the healed SWF‐ZAB lifted from one end. c) Schematic of the self‐healing mechanism of the SWF‐ZAB. d) Galvanostatic charge/discharge curves of the SWF‐ZAB in the pristine state and after multiple cutting/healing cycles. e) Photographs showing the SWF‐ZAB being cut and healed for five cycles (1‐3) and stably powering a digital watch for 1 day (4).

ZABs with self‐healing capabilities are desirable because they have extended service lives, lower raw material consumption, and lower environmental pollution. As shown in **Figure** 5a1,2, an SWF‐ZAB was cut in half with a knife to test its self‐healing properties. The destruction of its structural integrity caused the OCV of the SWF‐ZAB to immediately drop to 0 V (Figure 5b). Subsequently, the separated parts of the cut SWF‐ZAB were carefully reconnected and healed at room temperature (Figure 5a3). The mechanical properties and ionic conductivity of the PAM‐PEGMA‐IL ionogel were recovered through the reconstruction of the broken polymer network between the damaged surfaces (Figure 5c). As the PAM‐PEGMA‐IL ionogel healed, the separated electrodes adhered to the PAM‐PEGMA‐IL ionogel were reconnected (Figure S10a,b, Supporting Information), restoring conductivity to the previously broken electrodes (Figure 5c). The self‐healing of the PAM‐PEGMA‐IL ionogel and the reconnection of the electrodes allowed the SWF‐ZAB to recover its battery performance. As shown in Figure 5b, after 10 min of healing, the OCV curve of the healed SWF‐ZAB was similar to that of the pristine SWF‐ZAB, indicating the full recovery of battery performance. The electrodes of the healed SWF‐ZAB were firmly held together by the PAM‐PEGMA‐IL ionogel, which allowed the SWF‐ZAB to be lifted from one end without the electrode cracking (Figure 5a4) or OCV declining (Figure 5b). Furthermore, the repeatable self‐healing ability of the PAM‐PEGMA‐IL ionogel enabled the SWF‐ZAB to maintain its original battery performance even after multiple cutting/healing cycles in the same location. As shown in Figure 5d, the SWF‐ZAB was cut and allowed to heal for 10 min after 5, 10, 15, 20, and 25 h during the cycling process, followed by the resumption of discharging and charging. After five cycles of cutting and healing, the charge/discharge voltage gap of the SWF‐ZAB increased by only 0.03 V. Accordingly, the fading rate was 0.6% per cutting/healing cycle, indicating that the SWF‐ZABs had repeatable healing performance. To further demonstrate the importance of the healing capability of the SWF‐ZAB in extending their service life in practical applications, a SWF‐ZAB powering a digital watch was cut (Figure 5e1). After reconnecting the severed SWF‐ZAB for 10 s, the digital watch was powered again (Figure 5e2). Furthermore, even after five cutting and healing cycles, the SWF‐ZAB was still able to power the digital watch for over 24 h (Figure 5e3,e4).

## 3 Conclusion

Herein, a self‐healing PAM‐PEGMA‐IL ionogel was synthesized via the photopolymerization of AM and PEGMA in [Emim][DCA] with zinc acetate dihydrate for the fabrication of self‐healing and wide‐temperature flexible ZABs. The PAM‐PEGMA‐IL ionogel was cross‐linked by the abundant hydrogen bonds formed between amide groups. Moreover, the ionogel contained up to 80 wt.% [Emim][DCA] due to the formation of hydrogen bonds between PEG segments and [Emim][DCA]. The PAM‐PEGMA‐IL ionogel had high ionic conductivities of 4.1, 23.5, and 38.7 mS cm^−1^ at −40, 25, and 60 °C, good mechanical properties (toughness of 1.19 MJ m^−3^), a wide operating temperature range of −75–65 °C, and a high decomposition voltage of 2.43 V. The rational composition design of the PAM‐PEGMA‐IL ionogel resulted in the highest reported ionic conductivity and toughness among room‐temperature self‐healing ionogels containing 80 wt.% ILs. Furthermore, the dynamic nature of the hydrogen bonds in PAM‐PEGMA‐IL meant that this ionogel was capable of spontaneously restoring its damaged structural integrity and ionic conductivity even at sub‐zero temperatures. As a result, the SWF‐ZABs assembled with the PAM‐PEGMA‐IL ionogel demonstrated the longest cycling lives of 400, 340, and 275 h at −20, 25, and 40 °C compared to those of other reported hydrogel‐based ZABs. The SWF‐ZABs also exhibited good multi‐deformation stability as well as exceptional environmental stability, maintaining their battery performance even after exposure to extreme temperatures (−20 and 40 °C) and vacuum conditions for 10 days. The SWF‐ZABs were capable of powering wearable devices for extended periods of time and across a wide range of temperatures. In addition, inheriting the self‐healing capability of the PAM‐PEGMA‐IL ionogel, the SWF‐ZABs were capable of repeatedly healing their damaged battery performance, effectively increasing their reliability and service life. We believe that this promising application of self‐healing ionogels will provide a powerful tool in the creation of novel electrolytes for flexible rechargeable batteries with wide operating temperature ranges and long cycling lives.

## Conflict of Interest

The authors declare no conflict of interest.

## Supporting information

Supporting Information

Supplemental Movie 1

Supplemental Movie 2

## Data Availability

The data that support the findings of this study are available from the corresponding author upon reasonable request.
