# Supplementary material for: Self‐Healing Ionogel‐Enabled Self‐Healing and Wide‐Temperature Flexible Zinc‐Air Batteries with Ultra‐Long Cycling Lives
Source: Adv Sci (Weinh). 2024 Apr 3;11(25):2402193. doi: 10.1002/advs.202402193 (PMC11220675; doi:10.1002/advs.202402193)
Supplement: Supplementary file 1 — Supporting Information [file ADVS-11-2402193-s001.pdf]

ADVANCED SCIENCE
Open Access

Supporting Information

for *Adv. Sci.*, DOI 10.1002/advs.202402193

Self-Healing Ionogel-Enabled Self-Healing and Wide-Temperature Flexible Zinc-Air Batteries with Ultra-Long Cycling Lives

Hongli Li, Fuchang Xu, Yang Li and Junqi Sun*

Supporting Information

Self-Healing Ionogel-Enabled Self-Healing and Wide-Temperature Flexible Zinc-Air Batteries with Ultra-Long Cycling Lives

Hongli Li, Fuchang Xu, Yang Li and Junqi Sun*

State Key Laboratory of Supramolecular Structure and Materials, College of Chemistry, Jilin University, Changchun 130012, P.R. China.

* Corresponding author: yanglichem@jlu.edu.cn

Table of Content

1. Experimental Section 3
    1.1. Materials 3
    1.2. Characterization 3
    1.3. Electrochemical Performance of SWF-ZAB 3
    1.4. Fabrication of PAM-PEGMA-IL Ionogels 4
    1.5. Fabrication of PAM-PEGMA Hydrogels 4
    1.6. Fabrication of SWF-ZABs 4
    1.7. Low-Temperature Conductivity and Elasticity Test 5
    1.8. Informed Consent 5
2. Supplementary Figures 5
3. Supplementary Movies 10

1. Experimental Section

1.1. Materials

AM (98.0%), PEGMA (M_n = 480), and 1-hydroxycyclohexyl phenyl ketone (98.0%) were purchased from Tokyo Chemical Industry. Zinc acetate dihydrate (99.0%) and ammonium persulfate (99.0%) were purchased from Innochem. [Emim][DCA] (98.0%) was purchased from Lanzhou Institute of Chemical Physics. Pt/C, RuO_2, nickel foam (0.5 mm), and Nafion solution (DuPont, D520, 5 wt%) were purchased from Suzhou Sinero Technology Co., Ltd. All chemicals were used without further purification.

1.2. Characterization

The stress-strain tests were conducted using a 410R250 Tension Instrument (Test Resources Inc., USA) with a stretching speed of 50 mm min^{-1}. The FTIR spectra of the samples were obtained using a Bruker VERTEX 80 V FTIR spectrometer over the scanning range from 4000 cm^{-1} to 400 cm^{-1}. TGA was performed on the TA TGA550 instrument in a N_2 atmosphere, with a heating rate of 10 °C min^{-1} in the temperature range of 35 °C to 600 °C. The optical microscopy images were captured using an Olympus BX-51 microscope. Rheological measurement was performed using a TA HR-2 rheometer with an 8 mm parallel plate. The DSC measurement was conducted on a TA instruments Q200 in a N_2 atmosphere at a heating/cooling rate of 10 °C min^{-1}. The surface morphology of the Zn was characterized using a Regulus 8100 (Hitachi, Japan) field emission scanning electron microscopy. Optical images and videos were captured using a Canon PowerShot SX40 HS camera. The weight change of the electrolytes is calculated by the following equation:

$$\text{Weight change} = \frac{m}{m_0} \times 100\% \tag{1}$$

where m_0 is initial weight of the electrolyte, m is the weight of the electrolyte monitored over time.

1.3. Electrochemical Performance of SWF-ZAB

The ionic conductivity of the sample was measured using an electrochemical workstation (CHI 660E) through AC impedance between two stainless steel electrodes. The frequency range was from 1000 kHz to 0.1 Hz, with an amplitude of 5 mV. The ionic conductivity (δ) is calculated by the following equation:

$$\delta = \frac{L}{A \times R} \tag{2}$$

where L and A correspond to the thickness and area of the sample between the two stainless steel electrodes. R represents to the bulk resistance of the sample, which is the point where the AC impedance spectrum intersects with the real axis.

The ionic conductivity change of the electrolyte is calculated by the following equation:

$$\text{Ionic conductivity change} = \frac{\delta}{\delta_0} \times 100\% \tag{3}$$

where δ_0 is initial ionic conductivity of the electrolyte, δ is the ionic conductivity of the electrolyte monitored over time.

The galvanostatic charge/discharge and galvanostatic discharging performance was tested on a LAND-2100 automatic battery tester. The polarization performance of the SWF-ZAB was tested on an electrochemical workstation (CHI 660E).

The specific capacity (C) of the SWF-ZAB is calculated by the following equation:

$$C = \frac{\text{Discharge current} \times \text{Service hours}}{\text{Weight of consumed zinc}} \quad (4)$$

The power density (P) of the SWF-ZAB is calculated by the following equation:

$$P = I \times V \quad (5)$$

where I represents the discharging current density and V represents the corresponding SWF-ZAB voltage.

1.4. Fabrication of PAM-PEGMA-IL Ionogels

AM (0.70 g), PEGMA (0.30 g), zinc acetate dihydrate (0.44 g), and 1-hydroxycyclohexyl phenyl ketone (0.01g) were dissolved in [Emim][DCA] (4.00 g) and stirred until a homogeneous solution was obtained. The mixture was then degassed by sonication and allowed to polymerize for 30 minutes under UV light (365 nm, 250 W) to complete the gelation. The thicknesses of all PAM-PEGMA-IL ionogels were controlled to 0.5 mm.

1.5. Fabrication of PAM-PEGMA Hydrogels

A well-dispersed solution was obtained by mixing AM (0.70 g), PEGMA (0.30 g), zinc acetate dihydrate (0.44 g), and ammonium persulfate (0.01g) with H_2O (4.00 g). The mixture was then degassed by sonication and allowed to polymerize for 30 min at 60 °C to obtain the PAM-PEGMA hydrogel.

1.6. Fabrication of SWF-ZABs

The SWF-ZAB was fabricated by sandwiching the PAM-PEGMA-IL ionogel between a zinc foil and an air electrode. The air electrode was prepared as follows: Pt/C (20 mg), RuO_2 (20 mg), and Nafion solution (400 mg) were added in ethanol solution (2 mL, 50 wt%) to prepare a catalyst ink. The mixture was dispersed by sonication for 6 h. Subsequently, the slurry was coated onto nickel foam (0.5 mm) with a mass loading of 3 mg cm^{-2}, followed by drying at room temperature for 12 h. SWF-ZABs with different dimensions were prepared.

1.7. Low-Temperature Conductivity and Elasticity Test

As shown in Figure 1d and e, the LED bulb and the PAM-PEGMA-IL ionogel/PAM-PEGMA hydrogel were connected in series to a 10-V DC power supply. The setup was then placed on a glass Petridis filled with liquid nitrogen, with a distance of 1 cm between the ionogel/hydrogel and liquid nitrogen. The voltage applied to the LED bulb was approximately 2.5 V, as measured by the multimeter.

1.8. Informed Consent

No formal approval for the experiments related to wearable skin-technologies involving human subjects was required by Jilin University. Informed consent was obtained from human

subjects for all experiments prior to participation in the experiment. The right to privacy of human subjects has always been respected.

2. Supplementary Figures

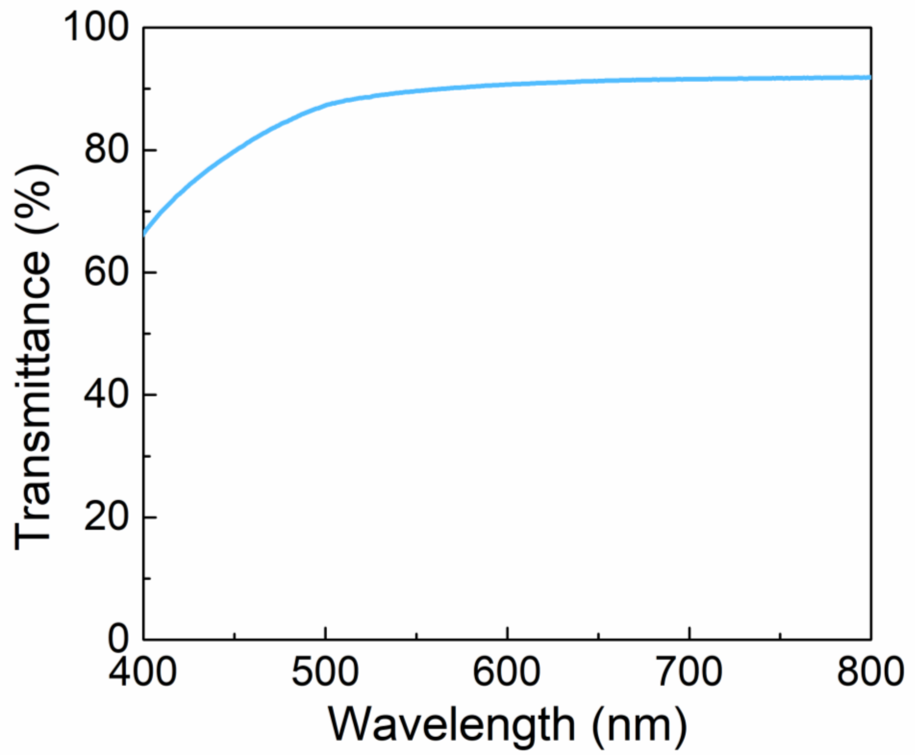

Figure S1. Transmittance spectrum of the PAM-PEGMA-IL ionogel with a thickness of 0.5 mm in the visible wavelength range.

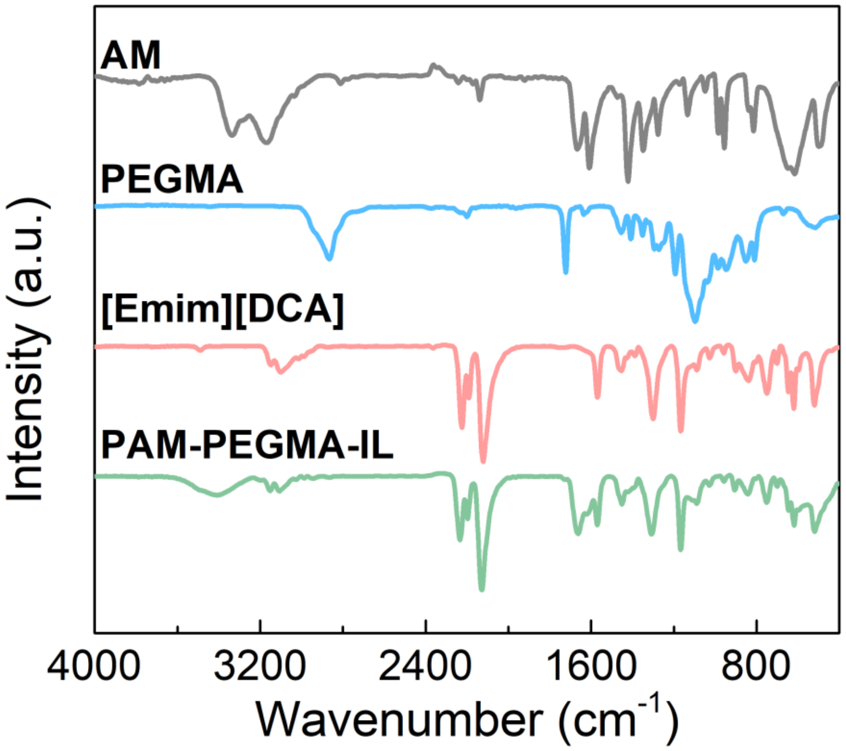

Figure S2. FTIR spectra of AM, PEGMA, [Emim][DCA], and PAM-PEGMA-IL ionogel.

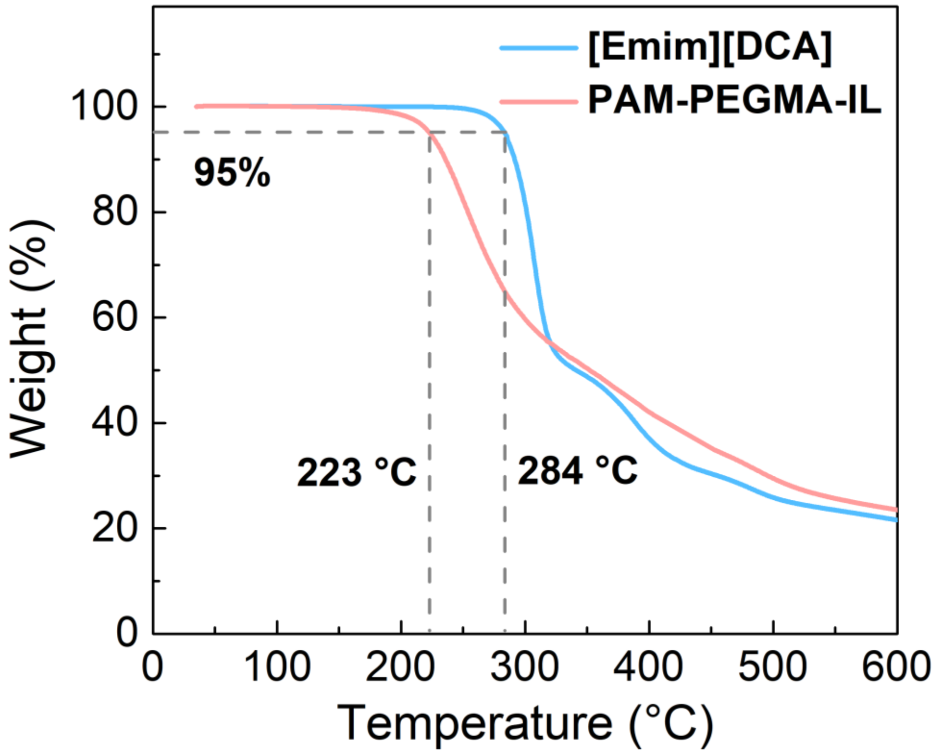

Figure S3. TGA curves of [Emim][DCA] and PAM-PEGMA-IL ionogel.

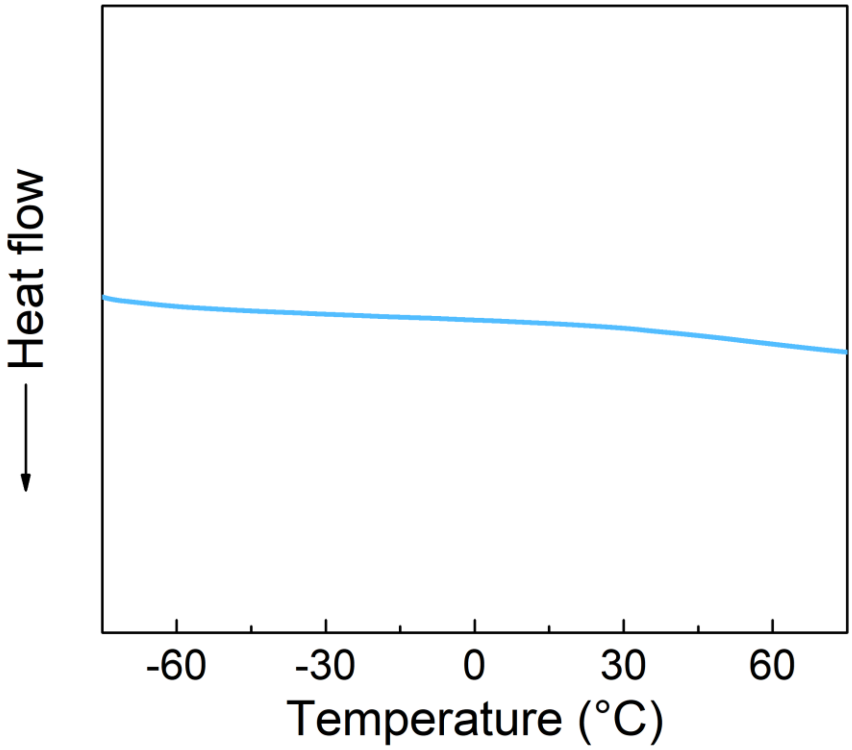

Figure S4. DSC curve of PAM-PEGMA-IL ionogel.

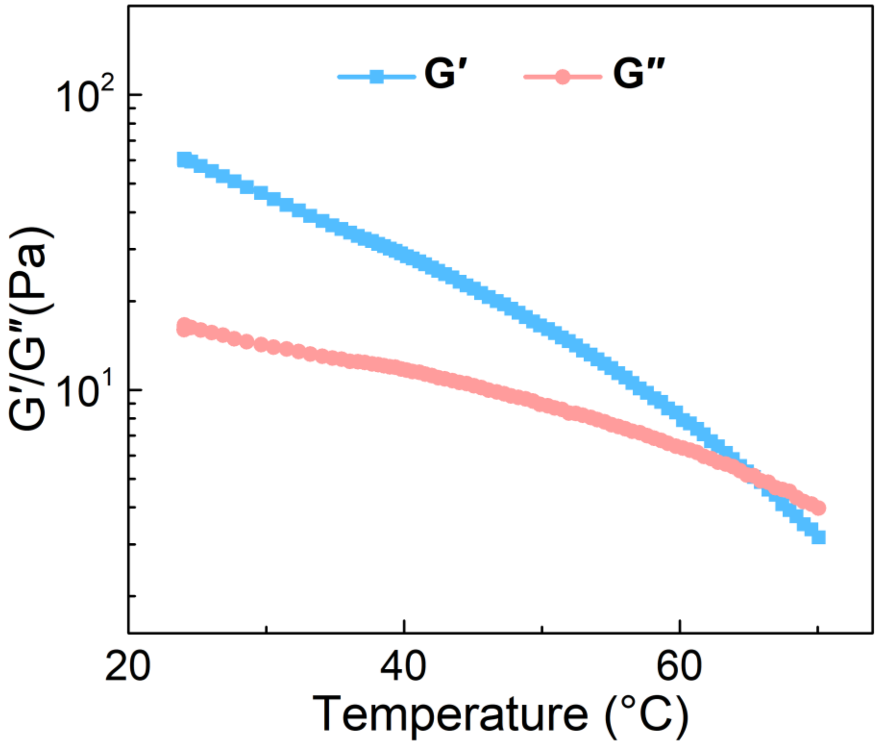

Figure S5. Temperature-dependent rheological curves of PAM-PEGMA-IL ionogel.

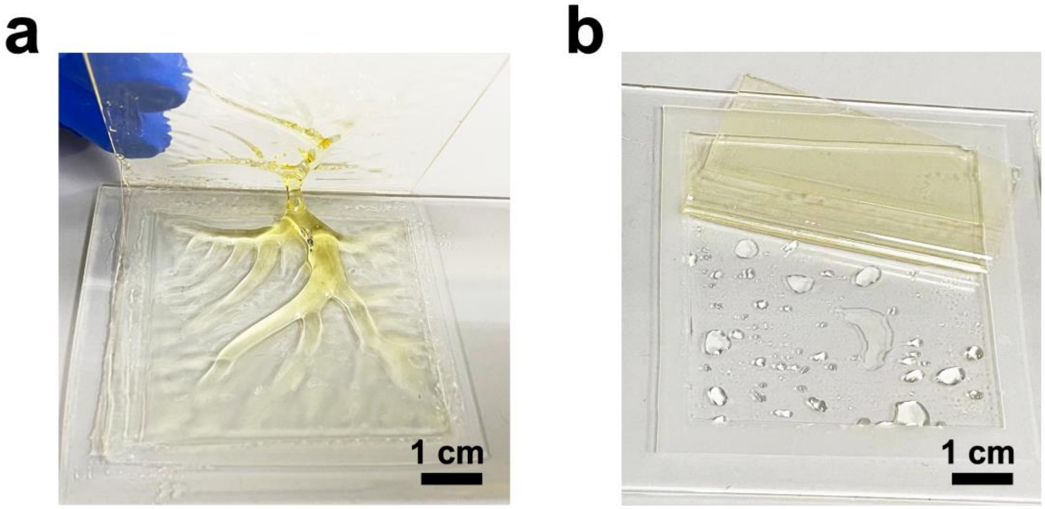

Figure S6. Photographs of (a) PAM-PEGMA-IL (m_{AM}:m_{PEGMA} = 6:4) and (b) PAM-PEGMA-IL (m_{AM}:m_{PEGMA} = 8:2) ionogels.

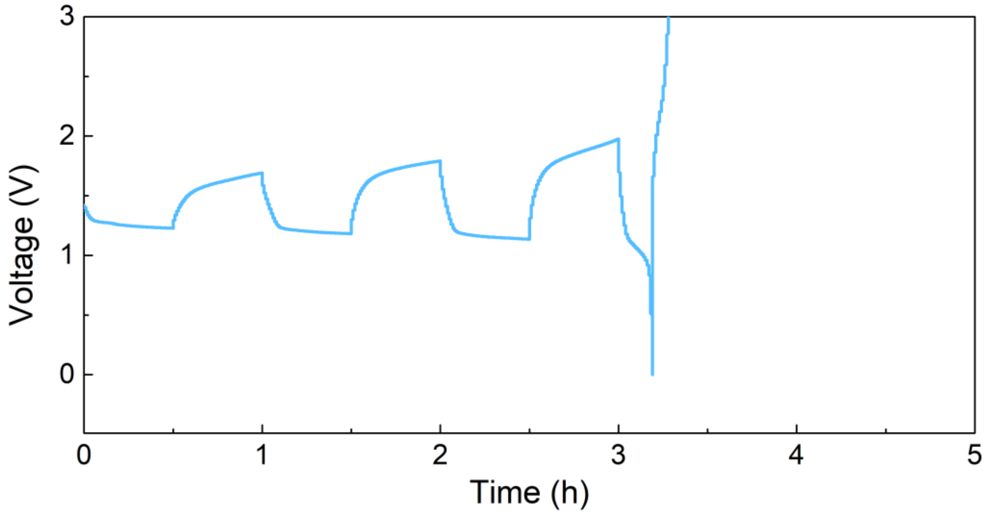

Figure S7. Galvanostatic charge/discharge curve of H-ZAB at 40 °C.

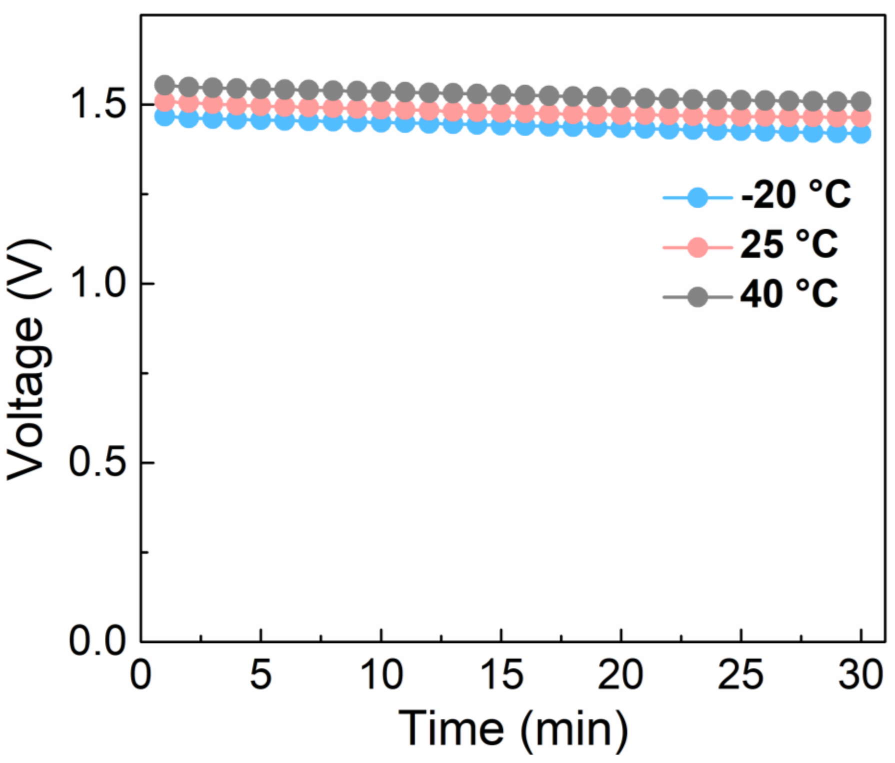

Figure S8. The OCVs of the SWF-ZAB at -20 °C, 25 °C, and 40 °C.

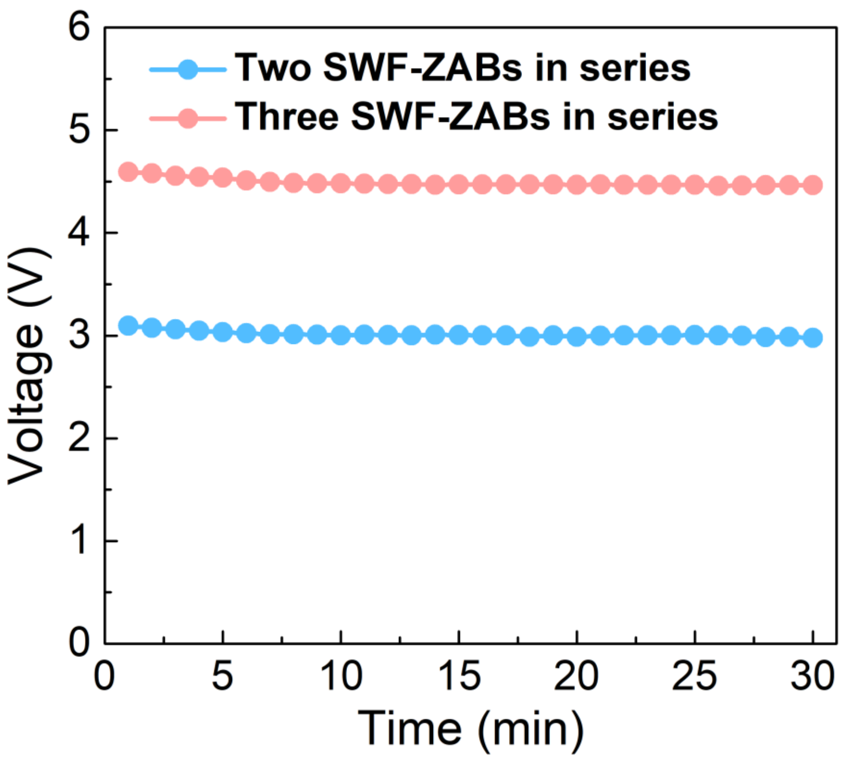

Figure S9. OCV curves of two and three SWF-ZABs connected in series.

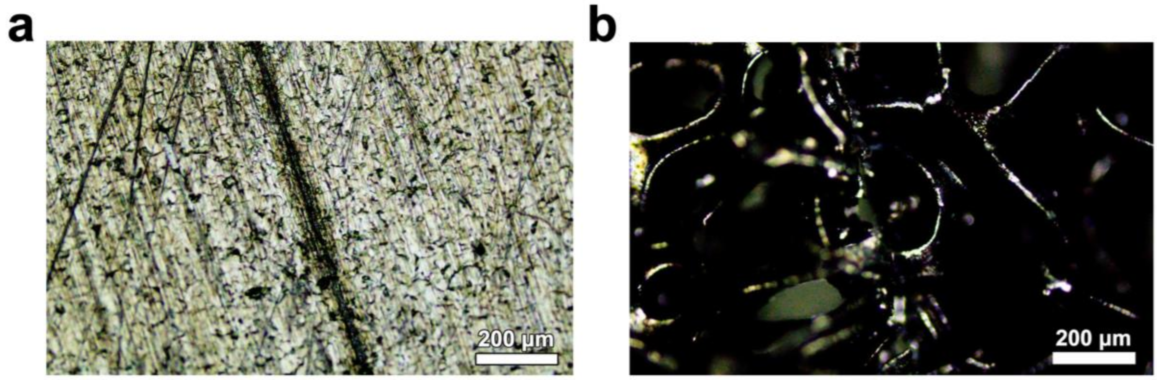

Figure S10. Optical microscopy images of the (a) Zn electrode and (b) air electrode of the healed SWF-ZAB.

3. Supplementary Movies

Movie S1. Movie featuring a PAM-PEGMA-IL ionogel being stretched to 200% strain at -60 °C while maintaining conductive properties.

Movie S2. Movie featuring a PAM-PEGMA hydrogel freezing and breaking under slight deformation at -60 °C.
